# Serial interval of SARS-CoV-2 was shortened over time by nonpharmaceutical interventions

**DOI:** 10.1126/science.abc9004

**Published:** 2020-07-21

**Authors:** Sheikh Taslim Ali, Lin Wang, Eric H. Y. Lau, Xiao-Ke Xu, Zhanwei Du, Ye Wu, Gabriel M. Leung, Benjamin J. Cowling

**Affiliations:** 1 WHO Collaborating Centre for Infectious Disease Epidemiology and Control, School of Public Health, Li Ka Shing Faculty of Medicine, The University of Hong Kong, Hong Kong Special Administrative Region, China.; 2 Department of Genetics, University of Cambridge, Cambridge CB2 3EH, UK.; 3 Mathematical Modelling of Infectious Diseases Unit, Institut Pasteur, UMR2000, CNRS, Paris 75015, France.; 4 College of Information and Communication Engineering, Dalian Minzu University, Dalian 116600, China.; 5 Department of Integrative Biology, University of Texas at Austin, Austin, TX 78705, USA.; 6 School of Journalism and Communication, Beijing Normal University, Beijing 100875, China.; 7 Computational Communication Research Center, Beijing Normal University, Zhuhai 519087, China.

## Abstract

Studies of novel coronavirus disease 2019 (COVID-19), which is caused by severe acute respiratory syndrome coronavirus 2 (SARS-CoV-2), have reported varying estimates of epidemiological parameters, including serial interval distributions—i.e., the time between illness onset in successive cases in a transmission chain—and reproduction numbers. By compiling a line-list database of transmission pairs in mainland China, we show that mean serial intervals of COVID-19 shortened substantially from 7.8 to 2.6 days within a month (9 January to 13 February 2020). This change was driven by enhanced nonpharmaceutical interventions, particularly case isolation. We also show that using real-time estimation of serial intervals allowing for variation over time provides more accurate estimates of reproduction numbers than using conventionally fixed serial interval distributions. These findings could improve our ability to assess transmission dynamics, forecast future incidence, and estimate the impact of control measures.

## From cough to splutter

In epidemiology, serial intervals are measured from when one infected person starts to show symptoms to when the next person infected becomes symptomatic. For any specific infection, the serial interval is assumed to be a fixed characteristic. Using valuable transmission pair data for coronavirus disease (COVID-19) in mainland China, Ali *et al.* noticed that the average serial interval changed as nonpharmaceutical interventions were introduced. In mid-January 2020, serial intervals were on average 7.8 days, whereas in early February 2020, they decreased to an average of 2.2 days. The more quickly infected persons were identified and isolated, the shorter the serial interval became and the fewer the opportunities for virus transmission. The change in serial interval may not only measure the effectiveness of infection control interventions but may also indicate rising population immunity.

*Science*, this issue p. 1106

## Teaser

The serial interval distribution of COVID-19 changes in response to interventions and offers a measure for effectiveness of control.

In December 2019, a novel coronavirus disease [coronavirus disease 2019 (COVID-19)], caused by severe acute respiratory syndrome coronavirus 2 (SARS-CoV-2), was first reported in Wuhan, China. It has since spread to more than 212 countries, causing more than 10 million confirmed cases and 500,000 deaths worldwide as of 30 June 2020 (*1*). Recent studies have suggested that several demographic and social factors can influence the transmission of COVID-19, including age- and gender-related differences in infection risk (*2*–*4*), reduced risk of infection as a result of intensive nonpharmaceutical interventions (NPIs) (e.g., isolation and social distancing) (*5*–*7*), and abrupt changes in social mixing patterns because of lockdowns and confinement (*8*–*10*). Serial interval, defined as the duration between the symptom-onset time of the infector and that of the infectee, is an essential metric for estimating many other key epidemiological parameters (e.g., reproduction number, generation time, and attack rate), which are used in turn to predict disease trends and health care demands (*11*). In early studies, before the availability of specific data on COVID-19, the serial interval distribution of COVID-19 was assumed to be similar to those of severe acute respiratory syndrome (SARS) or Middle East respiratory syndrome (MERS), with a mean >8 days (*12*, *13*). Once specific data became available on COVID-19 transmission pairs, several studies examined the serial interval distribution of COVID-19 in different locations, with estimates of the mean serial interval varying from 3.1 to 7.5 days (*6*, *14*–*21*). All of these studies have assumed that the timing of transmission events can be described by a single, stable distribution of serial intervals at different stages of an epidemic.

In fact, the serial interval depends on the incubation period, the profile of infectiousness after infection, and the variation in contact structure of the population (as explained in fig. S1) (*22*). The incubation period describes the biological process of disease progression and tends to follow a more similar distribution from one location to another, with minor variations resulting from social or cultural differences in how symptoms are perceived or reported. However, the profile of infectiousness over time can vary because of human behavior. Changes in contact patterns and the use of public health measures can reshape the timing of infection events by limiting successful contacts overall (e.g., social distancing) or after illness onset (e.g., case isolation). Interventions such as the isolation of confirmed and suspected cases, suspension of intra- and intercity travel, and different forms of social distancing were widely implemented in different Chinese cities. This provides an opportunity to study the temporal changes in the serial interval distribution and its association with NPIs. Here, we show that variation in the serial interval can occur and has important implications for the assessment of transmission dynamics and the impact of control measures.

We compiled a database of 1407 COVID-19 transmission pairs, in which symptom-onset dates and social relationships were available for both the infector and infectee of 677 transmission pairs [see table S1 for entire database (*23*) and supplementary materials for details]. Household and nonhousehold transmissions were identified on the basis of the information on social relationships (e.g., familial members of the same household, nonhousehold relatives, colleagues, classmates, friends, and other face-to-face contacts). The data were reconstructed from the publicly available reports of 9120 confirmed COVID-19 cases reported by 27 provincial and 264 urban health commissions in China outside Hubei province. Data from Hubei province were excluded because there was less reliable information on chains of transmission during the widespread community circulation of COVID-19; outside Hubei province, it was more straightforward to link connected cases and derive serial intervals. We focused on 677 transmission pairs with infectors having developed symptoms from 9 January through 13 February 2020. This 36-day period spans a series of key interventions related to the evolving epidemiology and transmission dynamics of COVID-19 in mainland China (*24*–*26*).

**Fig. 1:**
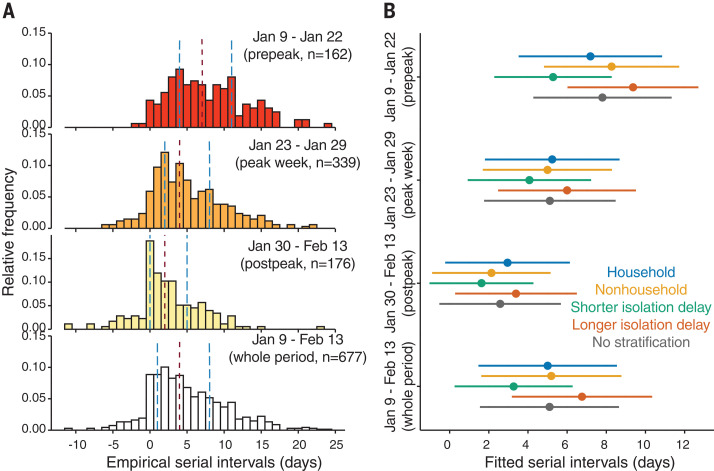
Serial intervals of SARS-CoV-2 substantially shortened over time in mainland China. (**A**) Empirical serial interval distributions. From top to bottom, transmission pairs were analyzed by selecting infectors who developed symptoms during 9 to 22 January 2020 (prepeak); 23 to 29 January 2020 (peak week); 30 January to 13 February 2020 (postpeak); and 9 January to 13 February 2020 (whole period), respectively. In each panel, vertical dashed lines in red and blue colors indicate the median and interquartile range (IQR), respectively. (**B**) Estimated serial interval distributions by fitting a normal distribution using MCMC. From top to bottom, each group of bars corresponds to the transmission pairs with infectors who developed symptoms during the prepeak (162 pairs), peak week (339 pairs), postpeak (176 pairs), and whole 36-day period (677 pairs), respectively. Colored dots and bars correspond to the transmission pairs within households (blue), outside households (yellow), with isolation delays shorter than the median isolation delay of each period (green), and with isolation delays longer than the median isolation delay of each period (orange), respectively. Dark gray bars correspond to transmission pairs with no stratification. Dots and bars indicate the estimated median and IQR, respectively.

We first calculated the number of transmission pairs in our database by the onset dates of infectors (fig. S3). Because many infectors (339) developed symptoms during 23 to 29 January 2020, we defined this 1-week period as the peak week, the previous 14-day period (9 to 22 January 2020) as the prepeak period, and the following 15-day period (30 January to 13 February 2020) as the postpeak period. We computed the serial interval as the number of days between the symptom-onset date of the infector and that of the infectee for each transmission pair. Empirical serial interval distributions for transmission pairs, counting from symptom onsets of the infectors during each period, indicate that the serial intervals shortened over time (Fig. 1A).

We estimated the serial interval distribution during each nonoverlapping period by fitting a normal distribution to the corresponding serial intervals data (supplementary materials). Analysis of all 677 transmission pairs revealed that the serial interval distribution had a mean of 5.1 [95% credibility interval (CrI): 4.7, 5.5] days and a standard deviation (SD) of 5.3 (95% CrI: 5.0, 5.6) days (table S2) overall, which is consistent with other recent studies (*16*, *21*, *27*). However, fitting to data of nonoverlapping periods of time revealed considerable variation in serial interval distributions (Fig. 1B). Before the peak, the mean and SD of serial intervals were estimated to be 7.8 (7.0, 8.6) days and 5.2 (4.7, 5.9) days, respectively. During the peak, the mean and SD reduced to 5.1 (4.6, 5.7) days and 5.0 (4.6, 5.4) days, respectively. After the peak, these estimates further shortened to 2.6 (1.9, 3.2) days and 4.6 (4.2, 5.1) days, respectively (table S2).

**Fig. 2:**
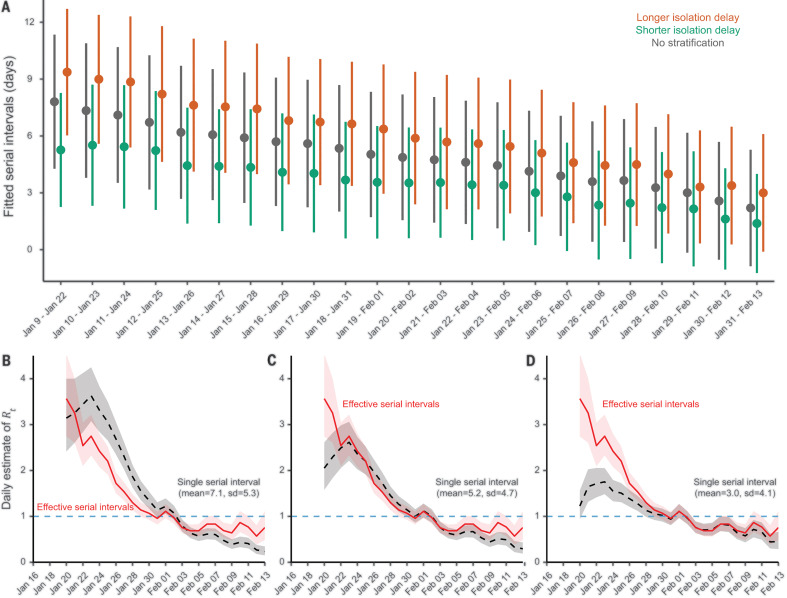
Real-time effective serial intervals and instantaneous reproduction number *R_t_*. (**A**) Estimated serial interval distribution for each 14-day running time window. Dark gray color indicates fitting data with no stratification, whereas green and orange indicate fitting data with isolation delay shorter and longer, respectively, than the median isolation delay of each running time window. Dots and bars indicate the estimated median and IQR, respectively. (**B** to **D**) Daily estimates of *R_t_* by using real-time effective serial interval distributions [as in (A)] versus using a single fixed serial interval distribution. Red curves and light pink shaded regions indicate the median and 95% CrI, respectively, of daily *R_t_* estimated using real-time effective serial interval distributions. Black dashed curves and light gray shaded regions indicate the median and 95% CrI, respectively, of daily *R_t_* estimated using a single serial interval distribution fixed, with a mean of 7.1 and SD of 5.3 days in (B), a mean of 5.2 and SD of 4.7 days in (C), and a mean of 3.0 and SD of 4.1 days in (D).

Next, we examined the real-time change in serial intervals by using a series of running time windows with fixed lengths of 10, 14, or 18 days (fig. S10). In contrast to the use of a constant distribution of serial intervals, our analysis suggests that serial intervals were gradually shortened over the study period (Fig. 2A), which is robust to alternative specifications of time windows (fig. S10). By fitting the transmission pairs data for each running time window by Markov chain Monte Carlo (MCMC) (Fig. 2A and table S3), we estimated that during the first 14-day period (9 to 22 January 2020), the serial intervals were longer on average [mean: 7.8 (95% CrI: 7.0, 8.6) days; SD: 5.2 (95% CrI: 4.7, 5.9) days]; whereas during the last 14 days (30 January to 13 February 2020), the serial intervals were much shorter on average [mean: 2.2 (1.5, 2.9) days; SD: 4.6 (4.1, 5.1) days]. Notably, the mean serial intervals were shortened by more than a factor of 3 over the 36-day period.

The transmission pair data also contain information for age, sex, household, and isolation delay (i.e., time duration from symptom onset to isolation) for most infectors. This allows for a granular stratification. Using either nonoverlapping or running time windows for data stratified by each of these factors, we find the same pattern of shorter serial intervals over time (Figs. 1B and 2A and tables S2 and S3). Therefore, we termed this changing serial interval the effective serial interval, which accounts for temporal changes caused by its potential driving factors. Notably, the length of effective serial intervals is positively associated with the length of isolation delay (Fig. 2A; figs. S5, S6, and S9; and tables S3 and S4), which accounts for the decreasing isolation delay over time (fig. S2). Therefore, early isolation (shorter than the median isolation delay) translates into shorter serial intervals [mean: 3.3 (2.7, 3.8) days; SD: 4.5 (4.1, 4.9) days], and delayed isolation (longer than the median isolation delay) is associated with longer serial intervals [mean: 6.8 (6.2, 7.3) days; SD: 5.3 (4.9, 5.7) days] (table S2). Stratification by age, gender, or household shows no clear differences in serial interval estimates. Our findings are robust to using alternative distributions (e.g., Gumbel distribution) for model fitting (fig. S11) and the infector-based approach (fig. S14).

Our probabilistic, individual-based simulated and regression models confirm that serial intervals are positively associated with isolation delay (section 5, supplementary materials). We found that the serial intervals become shorter on the basis of how much faster the infectors are isolated, regardless of when an infector starts to be infectious before symptom onset (fig. S5). In an individual-based simulation model with a mean generation time of 7.8 days, the simulated mean serial intervals are reduced from ~8.0 to ~1.2 days when the isolation delay is reduced from 10 to 0 days. We found through regression models that up to 51.5% of the variability in daily empirical serial interval can be explained by isolation delay and further improved by other NPI factors, which explain an additional 15.6 to 16.7% of the variability (table S5).

In practice, the time-varying serial interval may affect the estimation of epidemic parameters, including the transmissibility. The real-time transmissibility of an infectious disease is often characterized by the instantaneous reproduction number (*R_t_*), which is defined as the expected number of secondary infections caused by an infector on day *t*. The pathogen spreads when *R_t_* > 1 and is under control when *R_t_* < 1. To examine the effect of serial intervals on *R_t_*, we first obtained the daily number of cases on the basis of the onset dates of infectors and infectees among the 1407 transmission pairs (Fig. 2, B to D). By applying the statistical method developed by Cori *et al.* (*28*), we estimated *R_t_* for each day between 20 January and 13 February 2020. We noticed substantial differences in estimates of *R_t_* between using a single stable serial interval distribution and time-varying effective serial interval distributions. The magnitude of this difference is more prominent during the prepeak and postpeak periods than it is during the peak week when *R_t_* ≈ 1 (Fig. 2, B to D).

We observed that the serial interval for COVID-19 in mainland China was shortened by more than a factor of 3 in the 36 days between 9 January and 13 February 2020. This reduction was driven by intensive NPIs, particularly the reduction of the isolation delay period. Isolation of an infector 1 day earlier is expected to reduce the mean serial interval by 0.7 days. Thus, the serial interval was shortened by >3 days if infectors were rapidly isolated (Figs. 1B and 2A and tables S2 and S3). This is consistent with advocating isolation of cases and quarantining contacts within 1 day from symptom onset, which has been estimated to reduce COVID-19 transmission by 60% (*8*). We have not identified any substantial effects of gender or age of infectors on serial interval, but the NPIs were found to be significant for the transmission in communities rather than in households (table S5). Other studies (*15*, *20*) have estimated that the infectiousness of COVID-19 is greater at symptom onset. Although a short serial interval indicates that a substantial proportion of transmission events have occurred by the time symptoms are apparent (*14*), because of prolonged viral shedding (*14*, *29*, *30*) case isolation is still likely to reduce further transmission. Changes in the serial interval can therefore indicate effective implementation of specific transmission-reduction measures.

There are some limitations to our work. First, it is possible that there was recall bias on the onset of first symptoms in the line-list data; however, given the centralized pandemic response in mainland China, we expected that recall bias would not affect our main conclusions (figs. S12 and S13). Second, other factors may have influenced the reduction of effective serial intervals, as we can only explain up to 72% of the variance in observed serial intervals. Finally, our current transmission pair data do not contain variables about the potential exposure window of each case, which do not allow further inferences on the transmission potential.

Our results indicate that caution is needed when attempting to generalize estimates of the serial interval distribution to other places or to other periods of time in the same place, for example when estimating instantaneous reproductive numbers (Fig. 2, B to D). The real-time metric of effective serial intervals indicates that transmission models also need to account for the temporal variation in serial intervals as an epidemic proceeds. Effective serial intervals may provide better measurements of instantaneous transmissibility (*R_t_*)—because they include the effects of possible drivers of transmission—and could be helpful to policy-makers because they offer real-time information on the impact of public health measures.

## Supplementary Materials

science.sciencemag.org/content/369/6507/1106/suppl/DC1 Materials and Methods Figs. S1 to S14 Tables S1 to S5 References (*31*–*49*) MDAR Reproducibility Checklist

## Appendix

View/request a protocol for this paper from *Bio-protocol*.

## References

[1] World Health Organization (WHO), “Coronavirus disease 2019 (‎COVID-19)‎: Situation report – 162” (WHO, 2020); www.who.int/docs/default-source/coronaviruse/situation-reports/20200629-covid-19-sitrep-161.pdf?sfvrsn=74fde64e_2.

[2] M. U. G. Kraemer, C.-H. Yang, B. Gutierrez, C.-H. Wu, B. Klein, D. M. Pigott, Open COVID-19 Data Working Group, L. du Plessis, N. R. Faria, R. Li, W. P. Hanage, J. S. Brownstein, M. Layan, A. Vespignani, H. Tian, C. Dye, O. G. Pybus, S. V. Scarpino, The effect of human mobility and control measures on the COVID-19 epidemic in China. Science 368, 493–497 (2020). 10.1126/science.abb4218 32213647 PMC7146642

[3] C. Wenham, J. Smith, R. Morgan, Gender and COVID-19 Working Group, COVID-19: The gendered impacts of the outbreak. Lancet 395, 846–848 (2020). 10.1016/S0140-6736(20)30526-2 32151325 PMC7124625

[4] J. M. Jin, P. Bai, W. He, F. Wu, X.-F. Liu, D.-M. Han, S. Liu, J.-K. Yang, Gender Differences in Patients With COVID-19: Focus on Severity and Mortality. Front. Public Health 8, 152 (2020). 10.3389/fpubh.2020.00152 32411652 PMC7201103

[5] J. Hellewell, S. Abbott, A. Gimma, N. I. Bosse, C. I. Jarvis, T. W. Russell, J. D. Munday, A. J. Kucharski, W. J. Edmunds, Centre for the Mathematical Modelling of Infectious Diseases COVID-19 Working Group, S. Funk, R. M. Eggo, Feasibility of controlling COVID-19 outbreaks by isolation of cases and contacts. Lancet Glob. Health 8, e488–e496 (2020). 10.1016/S2214-109X(20)30074-7 32119825 PMC7097845

[6] L. Ferretti, C. Wymant, M. Kendall, L. Zhao, A. Nurtay, L. Abeler-Dörner, M. Parker, D. Bonsall, C. Fraser, Quantifying SARS-CoV-2 transmission suggests epidemic control with digital contact tracing. Science 368, eabb6936 (2020). 10.1126/science.abb6936 32234805 PMC7164555

[7] R. Armitage, L. B. Nellums, COVID-19 and the consequences of isolating the elderly. Lancet Public Health 5, e256 (2020). 10.1016/S2468-2667(20)30061-X 32199471 PMC7104160

[8] R. M. Anderson, H. Heesterbeek, D. Klinkenberg, T. D. Hollingsworth, How will country-based mitigation measures influence the course of the COVID-19 epidemic? Lancet 395, 931–934 (2020). 10.1016/S0140-6736(20)30567-5 32164834 PMC7158572

[9] J. R. Koo, A. R. Cook, M. Park, Y. Sun, H. Sun, J. T. Lim, C. Tam, B. L. Dickens, Interventions to mitigate early spread of SARS-CoV-2 in Singapore: A modelling study. Lancet Infect. Dis. 20, 678–688 (2020). 10.1016/S1473-3099(20)30162-6 32213332 PMC7158571

[10] K. Prem, Y. Liu, T. W. Russell, A. J. Kucharski, R. M. Eggo, N. Davies, Centre for the Mathematical Modelling of Infectious Diseases COVID-19 Working Group, M. Jit, P. Klepac, The effect of control strategies to reduce social mixing on outcomes of the COVID-19 epidemic in Wuhan, China: A modelling study. Lancet Public Health 5, e261–e270 (2020). 10.1016/S2468-2667(20)30073-6 32220655 PMC7158905

[11] M. A. Vink, M. C. Bootsma, J. Wallinga, Serial intervals of respiratory infectious diseases: A systematic review and analysis. Am. J. Epidemiol. 180, 865–875 (2014). 10.1093/aje/kwu209 25294601

[12] J. T. Wu, K. Leung, G. M. Leung, Nowcasting and forecasting the potential domestic and international spread of the 2019-nCoV outbreak originating in Wuhan, China: A modelling study. Lancet 395, 689–697 (2020). 10.1016/S0140-6736(20)30260-9 32014114 PMC7159271

[13] M. Chinazzi, J. T. Davis, M. Ajelli, C. Gioannini, M. Litvinova, S. Merler, A. Pastore Y Piontti, K. Mu, L. Rossi, K. Sun, C. Viboud, X. Xiong, H. Yu, M. E. Halloran, I. M. Longini Jr.., A. Vespignani, The effect of travel restrictions on the spread of the 2019 novel coronavirus (COVID-19) outbreak. Science 368, 395–400 (2020). 10.1126/science.aba9757 32144116 PMC7164386

[14] H. Nishiura, N. M. Linton, A. R. Akhmetzhanov, Serial interval of novel coronavirus (COVID-19) infections. Int. J. Infect. Dis. 93, 284–286 (2020). 10.1016/j.ijid.2020.02.060 32145466 PMC7128842

[15] H. Y. Cheng, S.-W. Jian, D.-P. Liu, T.-C. Ng, W.-T. Huang, H.-H. Lin, Taiwan COVID-19 Outbreak Investigation Team, Contact Tracing Assessment of COVID-19 Transmission Dynamics in Taiwan and Risk at Different Exposure Periods Before and After Symptom Onset. JAMA Intern. Med. 10.1001/jamainternmed.2020.2020 (2020). 10.1001/jamainternmed.2020.2020 32356867 PMC7195694

[16] Z. Du, X. Xu, Y. Wu, L. Wang, B. J. Cowling, L. A. Meyers, Serial Interval of COVID-19 among Publicly Reported Confirmed Cases. Emerg. Infect. Dis. 26, 1341–1343 (2020). 10.3201/eid2606.200357 32191173 PMC7258488

[17] Q. Li, X. Guan, P. Wu, X. Wang, L. Zhou, Y. Tong, R. Ren, K. S. M. Leung, E. H. Y. Lau, J. Y. Wong, X. Xing, N. Xiang, Y. Wu, C. Li, Q. Chen, D. Li, T. Liu, J. Zhao, M. Liu, W. Tu, C. Chen, L. Jin, R. Yang, Q. Wang, S. Zhou, R. Wang, H. Liu, Y. Luo, Y. Liu, G. Shao, H. Li, Z. Tao, Y. Yang, Z. Deng, B. Liu, Z. Ma, Y. Zhang, G. Shi, T. T. Y. Lam, J. T. Wu, G. F. Gao, B. J. Cowling, B. Yang, G. M. Leung, Z. Feng, Early Transmission Dynamics in Wuhan, China, of Novel Coronavirus-Infected Pneumonia. N. Engl. J. Med. 382, 1199–1207 (2020). 10.1056/NEJMoa2001316 31995857 PMC7121484

[18] J. M. Griffin, A. B. Collins, K. Hunt, D. McEvoy, M. Casey, A. W. Byrne, C. G. McAloon, A. Barber, E. A. Lane, S. J. More, A rapid review of available evidence on the serial interval and generation time of COVID-19. medRxiv 2020.05.08.20095075 [Preprint]. 11 May 2020. 10.1101/2020.05.08.20095075. 10.1101/2020.05.08.20095075 PMC7684810 33234640

[19] S. Ma, J. Zhang, M. Zeng, Q. Yun, W. Guo, Y. Zheng, S. Zhao, M. H. Wang, Z. Yang, Epidemiological parameters of coronavirus disease 2019: A pooled analysis of publicly reported individual data of 1155 cases from seven countries. medRxiv 2020.03.21.20040329 [Preprint]. 24 March 2020. 10.1101/2020.03.21.20040329. 10.1101/2020.03.21.20040329

[20] X. He, E. H. Y. Lau, P. Wu, X. Deng, J. Wang, X. Hao, Y. C. Lau, J. Y. Wong, Y. Guan, X. Tan, X. Mo, Y. Chen, B. Liao, W. Chen, F. Hu, Q. Zhang, M. Zhong, Y. Wu, L. Zhao, F. Zhang, B. J. Cowling, F. Li, G. M. Leung, Temporal dynamics in viral shedding and transmissibility of COVID-19. Nat. Med. 26, 672–675 (2020). 10.1038/s41591-020-0869-5 32296168

[21] Q. Bi, Y. Wu, S. Mei, C. Ye, X. Zou, Z. Zhang, X. Liu, L. Wei, S. A. Truelove, T. Zhang, W. Gao, C. Cheng, X. Tang, X. Wu, Y. Wu, B. Sun, S. Huang, Y. Sun, J. Zhang, T. Ma, J. Lessler, T. Feng, Epidemiology and transmission of COVID-19 in 391 cases and 1286 of their close contacts in Shenzhen, China: A retrospective cohort study. Lancet Infect. Dis. 20, 911–919 (2020). 10.1016/S1473-3099(20)30287-5 32353347 PMC7185944

[22] J. Zhang, M. Litvinova, Y. Liang, Y. Wang, W. Wang, S. Zhao, Q. Wu, S. Merler, C. Viboud, A. Vespignani, M. Ajelli, H. Yu, Changes in contact patterns shape the dynamics of the COVID-19 outbreak in China. Science 368, 1481–1486 (2020). 10.1126/science.abb8001 32350060 PMC7199529

[23] Lin, PDGLin/COVID19_EffSerialInterval_NPI: Serial interval of SARS-CoV-2 was shortened over time by non-pharmaceutical interventions, version v1.0, Zenodo (2020); 10.5281/zenodo.3940300. 10.5281/zenodo.3940300

[24] H. Tian, Y. Liu, Y. Li, C.-H. Wu, B. Chen, M. U. G. Kraemer, B. Li, J. Cai, B. Xu, Q. Yang, B. Wang, P. Yang, Y. Cui, Y. Song, P. Zheng, Q. Wang, O. N. Bjornstad, R. Yang, B. T. Grenfell, O. G. Pybus, C. Dye, An investigation of transmission control measures during the first 50 days of the COVID-19 epidemic in China. Science 368, 638–642 (2020). 10.1126/science.abb6105 32234804 PMC7164389

[25] K. Leung, J. T. Wu, D. Liu, G. M. Leung, First-wave COVID-19 transmissibility and severity in China outside Hubei after control measures, and second-wave scenario planning: A modelling impact assessment. Lancet 395, 1382–1393 (2020). 10.1016/S0140-6736(20)30746-7 32277878 PMC7195331

[26] A. Pan, L. Liu, C. Wang, H. Guo, X. Hao, Q. Wang, J. Huang, N. He, H. Yu, X. Lin, S. Wei, T. Wu, Association of Public Health Interventions With the Epidemiology of the COVID-19 Outbreak in Wuhan, China. JAMA 323, 1915–1923 (2020). 10.1001/jama.2020.6130 32275295 PMC7149375

[27] J. Zhang, M. Litvinova, W. Wang, Y. Wang, X. Deng, X. Chen, M. Li, W. Zheng, L. Yi, X. Chen, Q. Wu, Y. Liang, X. Wang, J. Yang, K. Sun, I. M. Longini Jr.., M. E. Halloran, P. Wu, B. J. Cowling, S. Merler, C. Viboud, A. Vespignani, M. Ajelli, H. Yu, Evolving epidemiology and transmission dynamics of coronavirus disease 2019 outside Hubei province, China: A descriptive and modelling study. Lancet Infect. Dis. 20, 793–802 (2020). 10.1016/S1473-3099(20)30230-9 32247326 PMC7269887

[28] A. Cori, N. M. Ferguson, C. Fraser, S. Cauchemez, A new framework and software to estimate time-varying reproduction numbers during epidemics. Am. J. Epidemiol. 178, 1505–1512 (2013). 10.1093/aje/kwt133 24043437 PMC3816335

[29] L. Zou, F. Ruan, M. Huang, L. Liang, H. Huang, Z. Hong, J. Yu, M. Kang, Y. Song, J. Xia, Q. Guo, T. Song, J. He, H.-L. Yen, M. Peiris, J. Wu, SARS-CoV-2 Viral Load in Upper Respiratory Specimens of Infected Patients. N. Engl. J. Med. 382, 1177–1179 (2020). 10.1056/NEJMc2001737 32074444 PMC7121626

[30] Y. Pan, D. Zhang, P. Yang, L. L. M. Poon, Q. Wang, Viral load of SARS-CoV-2 in clinical samples. Lancet Infect. Dis. 20, 411–412 (2020). 10.1016/S1473-3099(20)30113-4 32105638 PMC7128099

[31] X. K. Xu, X.-F. Liu, Y. Wu, S. T. Ali, Z. Du, P. Bosetti, E. H. Y. Lau, B. J. Cowling, L. Wang, Reconstruction of Transmission Pairs for novel Coronavirus Disease 2019 (COVID-19) in mainland China: Estimation of Super-spreading Events, Serial Interval, and Hazard of Infection. Clin. Infect. Dis. 10.1093/cid/ciaa790 (2020). 10.1093/cid/ciaa790 32556265 PMC7337632

[32] P. Trapman, F. Ball, J.-S. Dhersin, V. C. Tran, J. Wallinga, T. Britton, Inferring R0 in emerging epidemics-the effect of common population structure is small. J. R. Soc. Interface 13, 20160288 (2016). 10.1098/rsif.2016.0288 27581480 PMC5014060

[33] S. W. Park, D. Champredon, J. Dushoff, Inferring generation-interval distributions from contact-tracing data. J. R. Soc. Interface 17, 20190719 (2020). 10.1098/rsif.2019.0719 32574542 PMC7328397

[34] Q. H. Liu, M. Ajelli, A. Aleta, S. Merler, Y. Moreno, A. Vespignani, Measurability of the epidemic reproduction number in data-driven contact networks. Proc. Natl. Acad. Sci. U.S.A. 115, 12680–12685 (2018). 10.1073/pnas.1811115115 30463945 PMC6294899

[35] Y. Bai, L. Yao, T. Wei, F. Tian, D.-Y. Jin, L. Chen, M. Wang, Presumed Asymptomatic Carrier Transmission of COVID-19. JAMA 323, 1406–1407 (2020). 10.1001/jama.2020.2565 32083643 PMC7042844

[36] X. Pan, D. Chen, Y. Xia, X. Wu, T. Li, X. Ou, L. Zhou, J. Liu, Asymptomatic cases in a family cluster with SARS-CoV-2 infection. Lancet Infect. Dis. 20, 410–411 (2020). 10.1016/S1473-3099(20)30114-6 32087116 PMC7158985

[37] M. M. Arons, K. M. Hatfield, S. C. Reddy, A. Kimball, A. James, J. R. Jacobs, J. Taylor, K. Spicer, A. C. Bardossy, L. P. Oakley, S. Tanwar, J. W. Dyal, J. Harney, Z. Chisty, J. M. Bell, M. Methner, P. Paul, C. M. Carlson, H. P. McLaughlin, N. Thornburg, S. Tong, A. Tamin, Y. Tao, A. Uehara, J. Harcourt, S. Clark, C. Brostrom-Smith, L. C. Page, M. Kay, J. Lewis, P. Montgomery, N. D. Stone, T. A. Clark, M. A. Honein, J. S. Duchin, J. A. Jernigan, Public Health–Seattle and King County and CDC COVID-19 Investigation Team, Presymptomatic SARS-CoV-2 Infections and Transmission in a Skilled Nursing Facility. N. Engl. J. Med. 382, 2081–2090 (2020). 10.1056/NEJMoa2008457 32329971 PMC7200056

[38] T. Ganyani, C. Kremer, D. Chen, A. Torneri, C. Faes, J. Wallinga, N. Hens, Estimating the generation interval for coronavirus disease (COVID-19) based on symptom onset data, March 2020. Euro Surveill. 25, 2000257 (2020). 10.2807/1560-7917.ES.2020.25.17.2000257 32372755 PMC7201952

[39] W. E. Wei, Z. Li, C. J. Chiew, S. E. Yong, M. P. Toh, V. J. Lee, Presymptomatic Transmission of SARS-CoV-2 - Singapore, January 23-March 16, 2020. MMWR Morb. Mortal. Wkly. Rep. 69, 411–415 (2020). 10.15585/mmwr.mm6914e1 32271722 PMC7147908

[40] L. Tindale, M. Coombe, J. E. Stockdale, E. Garlock, W. Y. V. Lau, M. Saraswat, Y.-H. B. Lee, L. Zhang, D. Chen, J. Wallinga, C. Colijn, Transmission interval estimates suggest pre-symptomatic spread of COVID-19. medRxiv 2020.03.03.20029983 [Preprint]. 6 March 2020. . 10.1101/2020.03.03.20029983

[41] R. Wölfel, V. M. Corman, W. Guggemos, M. Seilmaier, S. Zange, M. A. Müller, D. Niemeyer, T. C. Jones, P. Vollmar, C. Rothe, M. Hoelscher, T. Bleicker, S. Brünink, J. Schneider, R. Ehmann, K. Zwirglmaier, C. Drosten, C. Wendtner, Virological assessment of hospitalized patients with COVID-2019. Nature 581, 465–469 (2020). 10.1038/s41586-020-2196-x 32235945

[42] A. T. Huang, B. Garcia-Carreras, M. D. T. Hitchings, B. Yang, L. Katzelnick, S. M. Rattigan, B. Borgert, C. Moreno, B. D. Solomon, I. Rodriguez-Barraquer, J. Lessler, H. Salje, D. S. Burke, A. Wesolowski, D. A. T. Cummings, A systematic review of antibody mediated immunity to coronaviruses: Antibody kinetics, correlates of protection, and association of antibody responses with severity of disease. medRxiv 2020.04.14.20065771 [Preprint]. 17 April 2020. . 10.1101/2020.04.14.20065771

[43] B. Rockx, T. Kuiken, S. Herfst, T. Bestebroer, M. M. Lamers, B. B. Oude Munnink, D. de Meulder, G. van Amerongen, J. van den Brand, N. M. A. Okba, D. Schipper, P. van Run, L. Leijten, R. Sikkema, E. Verschoor, B. Verstrepen, W. Bogers, J. Langermans, C. Drosten, M. Fentener van Vlissingen, R. Fouchier, R. de Swart, M. Koopmans, B. L. Haagmans, Comparative pathogenesis of COVID-19, MERS, and SARS in a nonhuman primate model. Science 368, 1012–1015 (2020). 10.1126/science.abb7314 32303590 PMC7164679

[44] M. Lipsitch, T. Cohen, B. Cooper, J. M. Robins, S. Ma, L. James, G. Gopalakrishna, S. K. Chew, C. C. Tan, M. H. Samore, D. Fisman, M. Murray, Transmission dynamics and control of severe acute respiratory syndrome. Science 300, 1966–1970 (2003). 10.1126/science.1086616 12766207 PMC2760158

[45] K. M. Gostic, L. McGough, E. Baskerville, S. Abbott, K. Joshi, C. Tedijanto, R. Kahn, R. Niehus, J. A. Hay, P. M. De Salazar, J. Hellewell, S. Meakin, J. Munday, N. Bosse, K. Sherratt, R. M. Thompson, L. F. White, J. Huisman, J. Scire, S. Bonhoeffer, T. Stadler, J. Wallinga, S. Funk, M. Lipsitch, S. Cobey, Practical considerations for measuring the effective reproductive number, Rt. medRxiv 2020.06.18.20134858 [Preprint]. 23 June 2020. . 10.1101/2020.06.18.20134858

[46] J. Wallinga, P. Teunis, Different epidemic curves for severe acute respiratory syndrome reveal similar impacts of control measures. Am. J. Epidemiol. 160, 509–516 (2004). 10.1093/aje/kwh255 15353409 PMC7110200

[47] C. Fraser, Estimating individual and household reproduction numbers in an emerging epidemic. PLOS ONE 2, e758 (2007). 10.1371/journal.pone.0000758 17712406 PMC1950082

[48] Z. Li, Q. Chen, L. Feng, L. Rodewald, Y. Xia, H. Yu, R. Zhang, Z. An, W. Yin, W. Chen, Y. Qin, Z. Peng, T. Zhang, D. Ni, J. Cui, Q. Wang, X. Yang, M. Zhang, X. Ren, D. Wu, X. Sun, Y. Li, L. Zhou, X. Qi, T. Song, G. F. Gao, Z. Feng, China CDC COVID-19 Emergency Response Strategy Team, Active case finding with case management: The key to tackling the COVID-19 pandemic. Lancet 396, 63–70 (2020). 10.1016/S0140-6736(20)31278-2 32505220 PMC7272157

[49] P. Wu, X. Hao, E. H. Y. Lau, J. Y. Wong, K. S. M. Leung, J. T. Wu, B. J. Cowling, G. M. Leung, Real-time tentative assessment of the epidemiological characteristics of novel coronavirus infections in Wuhan, China, as at 22 January 2020. Euro Surveill. 25, 2000044 (2020). 10.2807/1560-7917.ES.2020.25.3.2000044 31992388 PMC6988272

